# Respiratory symptoms in relation to residential coal burning and environmental tobacco smoke among early adolescents in Wuhan, China: a cross-sectional study

**DOI:** 10.1186/1476-069X-3-14

**Published:** 2004-12-07

**Authors:** Päivi M Salo, Jiang Xia, C Anderson Johnson, Yan Li, Grace E Kissling, Edward L Avol, Chunhong Liu, Stephanie J London

**Affiliations:** 1 Epidemiology Branch, National Institute of Environmental Health Sciences, National Institutes of Health, MD A3-05, PO Box 12233, Research Triangle Park, NC 27709, USA; 2 Wuhan Public Health and Anti-Epidemic Station, No. 24 N. Jianghan Road, Wuhan, Hubei 430022, China; 3 Institute for Health Promotion & Disease Prevention Research, USC Keck School of Medicine, 1000 South Fremont Ave., Unit 8, Alhambra, CA 91803, USA; 4 Wuhan Health Bureau, 2 YiYuan Road, Wuhan, Hubei 430014, China; 5 Biostatistics Branch, National Institute of Environmental Health Sciences, National Institutes of Health, MD A3-03, PO Box 12233, Research Triangle Park, NC 27709, USA; 6 Department of Occupational & Environmental Health, USC Keck School of Medicine, CHP 236, 1540 Alcazar St., Los Angeles, CA 90089, USA

## Abstract

**Background:**

Cigarette smoking and coal burning are the primary sources of indoor air pollution in Chinese households. However, effects of these exposures on Chinese children's respiratory health are not well characterized.

**Methods:**

Seventh grade students (N = 5051) from 22 randomly selected schools in the greater metropolitan area of Wuhan, China, completed an in-class self-administered questionnaire on their respiratory health and home environment.

**Results:**

Coal burning for cooking and/or heating increased odds of wheezing with colds [odds ratio (OR) = 1.57, 95% confidence interval (CI): 1.07–2.29] and without colds (OR = 1.44, 95% CI: 1.05–1.97). For smoking in the home, the strongest associations were seen for cough (OR = 1.74, 95% CI: 1.17–2.60) and phlegm production (OR = 2.25, 95% CI: 1.36–3.72) without colds among children who lived with two or more smokers.

**Conclusions:**

Chinese children living with smokers or in coal-burning homes are at increased risk for respiratory impairment. While economic development in China may decrease coal burning by providing cleaner fuels for household energy use, the increasing prevalence of cigarette smoking is a growing public health concern due to its effects on children. Adverse effects of tobacco smoke exposure were seen despite the low rates of maternal smoking (3.6%) in this population.

## Background

Residential coal burning and cigarette smoking are the most common sources of indoor air pollution in Chinese households [1-3]. Although use of coal stoves and smoking have been linked to respiratory morbidity among adult populations in mainland China [1,4-6], little is known about how these exposures affect Chinese children's respiratory health. Children's developing lungs are especially vulnerable to indoor air pollution because children spend much of their time indoors at home [7,8].

Coal has been widely used for cooking and heating in China [1,9]. Domestic coal stoves and boilers produce high indoor concentrations of sulfur dioxide (SO_2_), carbon monoxide (CO), particulate matter, and other pollutants [2,3,10,11]. Some studies suggest that indoor concentrations of emitted pollutants may exceed international guidelines and national ambient air pollution standards in China [1,2].

An increasing proportion of Chinese children are exposed to tobacco smoke because smoking prevalence in China has increased rapidly, especially among men, during the last decades [1,12]. Over two thirds of the Chinese population is currently exposed to environmental tobacco smoke (ETS) in the home [12]. Literature from Western populations has generally shown that maternal smoking is more strongly associated with children's respiratory symptoms than paternal smoking [13,14]. China provides a unique opportunity to examine effects of parental smoking because prevalence of smoking remains very low among women.

We examined the associations between respiratory symptoms and exposure to residential coal burning and environmental tobacco smoke in a cross-sectional study of seventh grade students in the greater metropolitan area of Wuhan, China.

## Methods

### Participants and study procedure

In the spring of 1999, 5231 seventh grade students at 22 public schools in and around Wuhan, China were invited to complete a self-administered questionnaire on respiratory symptoms and home environment. Two schools were randomly selected from each of the 11 administrative units governed by the city of Wuhan, the capital of Hubei Province. The resulting schools included 14 schools from urban (53.0% of the students), three schools from suburban (25.1%), and five schools from rural areas (21.9%). Of the 5231 students, 5051 (97 %) provided parental consent and completed the questionnaire in class with study staff in attendance. Before completing the questions students viewed a video demonstrating wheezing [15]. The study protocol was approved by the Institutional Review Boards at the Wuhan Public Health and Anti-Epidemic Station and the National Institute of Environmental Health Sciences.

### Questionnaire data

The questionnaire included questions on respiratory health and potential risk factors such as exposure to cooking and heating fuels, smokers in the home, and personal smoking. We incorporated respiratory health items from a standardized questionnaire (ATS-DLD-78-C) translated into Chinese for a previous study in Wuhan and other Chinese cities [16]. Our primary outcome measures were responses to questions regarding respiratory symptoms in the past 12 months. We asked whether children had cough and/or phlegm production almost everyday during the past 12 months, with and without colds. Additionally, we asked whether children had wheezing over the past 12 months, with and without colds.

We assessed exposure to residential coal burning by questions defining the types of fuels used for cooking and heating in the child's home. We combined information on cooking and heating with coal into a single variable with the following categories: no coal stove, coal stove used only for heating, coal stove used only for cooking, and coal stove used for both cooking and heating. To assess environmental tobacco smoke exposure, we asked the child to list all household members and indicate whether each person currently smoked. We created three exposure categories: no smokers in the home, one smoker (1) in the home, and two or more smokers (2 +) in the home. Personal smoking was not considered in the analysis because of very low prevalence (0.6%).

### Statistical analysis

We calculated prevalence odds ratios (95% confidence intervals) for each of the six outcome measures (cough, phlegm production and wheezing in the past 12 months, each with colds and without colds) by unconditional logistic regression (Proc Genmod in SAS System for Windows, Version 8.01). Although the odds ratio is the most common measure of association in cross-sectional studies [17], divergence between odds ratios and risk ratios increases as the outcome becomes more common [18,19]. However, we present odds ratios as our effect measures to estimate the associations between respiratory symptoms and residential exposures (coal burning and environmental tobacco smoke); using the log-binomial model (Proc Genmod in SAS System for Windows, Version 8.01) [20] to estimate prevalence proportion ratios for the outcomes did not alter any of our conclusions.

We excluded 521 subjects with missing data on any of the outcome or exposure variables leaving 4,530 subjects for the analysis. The following covariates were considered as potential confounders or modifying factors within the logistic models: child's gender, presence of animals in the household, presence of pests (cockroaches, ants, rodents), crowding in the household, presence of older siblings, parental asthma, physical activity, living area (school district), and time spent indoors and outdoors. To account for variation due to the type of neighborhood the children lived in, we included school district (22 districts) in the models using CLASS and REPEATED statements within Proc Genmod in SAS. The models reported here are adjusted for coal use, smokers in the home, school district, and child's sex because inclusion of the other variables did not appreciably change the associations.

## Results

Characteristics of the study population are presented in Table 1. The mean age of the seventh grade students was 13.6 years (SD = 0.7 years). The majority of the students (94.2%) were life-long residents of the Wuhan area. Although 7.1% of the students reported wheezing without colds, doctor-diagnosed asthma was relatively uncommon in this population (3.2%). Coal was used for cooking and/or heating in almost half of the homes. Few children smoked (0.6%), but 73.2% of the students lived with household members who smoked. The prevalence of ETS exposure was similar across the study area (74.5% in urban areas, 70.2% in suburban areas, and 73.5% in rural areas). Fathers (69.1%) were much more likely to smoke than mothers (3.6%).

**Table 1:** Characteristics of the study population of 4530 students at 22 schools in greater Wuhan, China

Characteristic	%
**Subjects**	
Age (mean, SD) in years	13.6, 0.7
Gender	
Male Female	52.5 47.5

**Respiratory symptoms**	
Wheezing with colds	19.4
Wheezing without colds	7.1
Bringing up phlegm with colds	16.7
Bringing up phlegm without colds	5.7
Coughing with colds	24.7
Coughing without colds	4.5

**Exposures**	
Smokers in child's household	
No smokers 1 smoker 2+ smokers Father smokes Mother smokes Personal smoking by students	26.8 62.3 10.9 69.1 3.6 0.6
	
Exposure to coal burning	
No coal use Coal used only for heating Coal used only for cooking Coal used for cooking and heating	54.2 8.8 25.9 11.1

After adjusting for gender, ETS, and living area, residential coal burning was primarily associated with wheezing in the past 12 months (Table 2). For those who used coal only for cooking or only for heating, wheezing was more strongly associated with cooking. However, the association between coal use and recent wheezing tended to strengthen when coal was used for both cooking and heating (OR = 1.78, 95% CI: 1.08–2.91 for wheezing with colds; OR = 1.57, 95% CI: 0.94–2.64 for wheezing without colds).

**Table 2:** Respiratory symptoms in relation to residential coal burning

	**Cough with colds**				**Cough without colds**			
	No	Yes			No	Yes		
	
**Exposure**	N	N	OR*	(95% CI)	N	N	OR*	(95% CI)
Total	3413	1117			4327	203		
**Coal use**								
No	1833	622	1.00		2347	108	1.00	
Yes	1580	495	0.92	(0.76,1.11)	1980	95	1.03	(0.80,1.33)
Heating	300	99	0.96	(0.76,1.22)	381	18	1.02	(0.67,1.55)
Cooking	926	249	0.79	(0.67,0.94)	1120	55	1.04	(0.74,1.46)
Both	354	147	1.22	(0.93,1.59)	479	22	0.99	(0.66,1.49)
	**Phlegm with colds**				**Phlegm without colds**			
	No	Yes			No	Yes		
	
	N	N	OR*	(95% CI)	N	N	OR*	(95% CI)
	
Total	3772	758			4274	256		
**Coal use**								
No	2051	404	1.00		2315	140	1.00	
Yes	1721	354	1.04	(0.90,1.20)	1959	116	0.96	(0.75,1.23)
Heating	331	68	1.04	(0.86,1.27)	376	23	1.02	(0.62,1.66)
Cooking	994	181	0.92	(0.76,1.10)	1114	61	0.86	(0.64,1.16)
Both	396	105	1.34	(1.05,1.73)	469	32	1.12	(0.81,1.54)
	**Wheeze with colds**				**Wheeze without colds**			
	No	Yes			No	Yes		
	
	N	N	OR*	(95% CI)	N	N	OR*	(95% CI)
	
Total	3652	878			4210	320		
**Coal use**								
No	2058	397	1.00		2309	146	1.00	
Yes	1594	481	1.57	(1.07,2.29)	1901	174	1.44	(1.05,1.97)
Heating	329	70	1.10	(0.76,1.57)	368	31	1.35	(0.86,2.15)
Cooking	892	283	1.66	(1.01,2.73)	1077	98	1.42	(1.05,1.92)
Both	373	128	1.78	(1.08,2.91)	456	45	1.57	(0.94,2.64)

* Odds ratios (OR) adjusted for gender, ETS, and school district. Dichotomous and multilevel odds ratios are computed in separate models.

After adjusting for gender, coal use, and living area, living with smokers (Table 3) was significantly associated with chronic cough and phlegm production in the past 12 months. The strongest associations were seen for cough (OR = 1.74, 95% CI: 1.17–2.60) and phlegm production (OR = 2.25, 95% CI: 1.36–3.72) without colds among children who lived with two or more smokers. Living with smokers was not appreciably associated with wheezing.

**Table 3:** Respiratory symptoms in relation to living with smokers

	**Cough with colds**				**Cough without colds**			
	No	Yes			No	Yes		
	
**Exposure**	N	N	OR*	(95% CI)	N	N	OR*	(95% CI)
Total	3413	1117			4327	203		
**Smokers in the home**								
No	954	259	1.00		1165	48	1.00	
Yes	2459	858	1.29	(1.05,1.58)	3162	155	1.19	(0.86,1.65)
1 smoker	2105	717	1.26	(1.02,1.55)	2700	122	1.10	(0.77,1.57)
2+ smokers	354	141	1.47	(1.11,1.95)	462	33	1.74	(1.17,2.60)

	**Phlegm with colds**				**Phlegm without colds**			
	No	Yes			No	Yes		
	
	N	N	OR*	(95% CI)	N	N	OR*	(95% CI)
	
Total	3772	758			4274	256		
**Smokers in the home**								
No	1036	177	1.00		1164	49	1.00	
Yes	2736	581	1.24	(1.08,1.43)	3110	207	1.60	(1.11,2.29)
1 smoker	2327	495	1.25	(1.09,1.43)	2657	165	1.49	(1.04,2.14)
2+ smokers	409	86	1.23	(0.92,1.64)	453	42	2.25	(1.36,3.72)

	**Wheeze with colds**				**Wheeze without colds**			
	No	Yes			No	Yes		
	
	N	N	OR*	(95% CI)	N	N	OR*	(95% CI)
	
Total	3652	878			4210	320		
**Smokers in the home**								
No	993	220	1.00		1125	88	1.00	
Yes	2659	658	1.11	(0.93,1.31)	3085	232	0.96	(0.74,1.25)
1 smoker	2265	557	1.10	(0.93,1.30)	2619	203	0.99	(0.75,1.30)
2+ smokers	394	101	1.13	(0.85,1.49)	466	29	0.78	(0.45,1.37)

* Odds ratios (OR) adjusted for gender, coal use, and school district. Dichotomous and multilevel odds ratios are computed in separate models.

## Discussion

Domestic coal use and exposure to ETS in the home were both associated with adverse respiratory effects in this population of Chinese adolescents. Coal burning was associated with increased wheezing, whereas living with smokers was associated with increased cough and phlegm production.

Coal burning produces high concentrations of particulate matter, SO_2_, and other pollutants [2,3,11]. Exposure to these pollutants may impair clearance mechanisms, and lead to airway inflammation [21,22]. Decreased pulmonary function has been associated with exposure to particulate matter and SO_2 _in several air pollution studies during the past decades [21]. Although residential coal burning has been linked to decreased pulmonary function and asthma among children [23-25], conflicting data exist. In two European studies, domestic coal burning has been associated with lower risk for childhood asthma and allergic diseases [26,27]. The findings in these two studies, however, may reflect some early life or other lifestyle factors related to coal use in Europe.

In our study, residential coal burning was predominantly associated with wheezing. Coal cooking was a stronger risk factor for wheezing than was coal heating. This may be explained by relatively low heating use in the Wuhan area, whereas cooking is a year around activity. The greater association with coal use for both cooking and heating may suggest an exposure-dependent relationship.

Although wheezing is often closely related to asthma, coal use was not positively associated with asthma diagnosis (data not shown) in this population. The majority of the diagnosed asthmatics (76.4%) lived in urban areas, where prevalence of coal use was lower than in non-urban areas. The diagnostic ascertainment of asthma most likely was greater in the urban than in the rural areas.

The harmful effects of ETS in children, primarily from living with smokers, have been widely studied [14,28-31]. In general, evidence that ETS causes cough, phlegm, and wheezing has not been as strong for school-aged children as it has been for infants and preschool children [28]. There are few data among Chinese populations where smoking behavior differs from Western populations. *In utero *exposure, via maternal smoking, that is believed to contribute to adverse effects of ETS in children [32,33] is uncommon in China. Thus, it is of interest that in this group of middle school children, where maternal and personal smoking were low, exposure to ETS in the home was clearly associated with chronic cough and phlegm production, with and without colds.

Our results indicated an exposure dependent response to ETS; having two or more smokers in the household increased the odds of cough and phlegm production compared to having only one smoker in the household. We did not find strong evidence suggesting modifying effects by gender, although the effect of ETS on persistent cough without colds was more pronounced among boys than girls (data not shown). Exposure levels may be influenced by time-activity patterns that can differ by gender. Boys may be more likely to spend time in close proximity with their smoking fathers or male relatives than girls.

Mechanisms responsible for the respiratory effects of ETS have been proposed in the literature [28]. In addition to decreased mucociliary clearance and goblet cell hypertrophy/hypersecretion, local and central nervous system components are thought be involved in cough and phlegm production [28,34]. Although exposure to ETS may affect childhood lung growth and result in lower pulmonary function [14,35], wheezing was not appreciably related to the presence of smokers in our study. Genetic susceptibility may influence the effects of ETS on bronchial obstruction. For example, parental atopy was found to modify the effects of ETS on bronchial obstruction and asthma considerably in a Norwegian birth cohort study [36]. However, we were unable to examine potential interactions between family history and ETS in relation to atopic illness in our population because, consistent with previously published data on Chinese children [37,38], the prevalence of asthma (3.2%) and hay fever (1.8%) was very low.

In general, our findings agree with available data on Chinese children's respiratory health [16,23]. However, residential exposures in the current study were more selectively associated with the respiratory symptoms than in previous studies. This may reflect differences in the study settings. In the previous studies [16,23], for example, most of the children were younger in age than in the current study. Prevalence of symptoms and factors associated with childhood respiratory symptoms may differ between different age groups [39]. It is also possible that using students rather than parents as a source of information on child's symptoms may contribute to the observed differences.

Exposure to indoor air pollutants is not only influenced by the source strength and other emission characteristics, but also by air exchange rates. A recent study showed that ventilation could modify effects between respiratory health outcomes and indoor air pollutants [40]. In that study, the modifying effects were found most relevant when air exchange rates were low. Residences in Wuhan, however, were not energy-efficiently built [16]. Air conditioning was uncommon, and most of the homes, both in urban and non-urban areas, relied on natural ventilation. In this study, we were unable to evaluate the effects of ventilation rates, because we did not collect detailed information on ventilation practices. We thought that children would not be able to give this information accurately.

The composition of pollutants produced by residential coal burning and smoking can be highly variable, but both exposures contribute substantially to inhalable and respirable particulate matter in indoor environments [2,3,41]. Existing data suggests that coal burning and smoking may have synergistic effects on respiratory symptoms [5]. In our data, we did not find consistent evidence of interaction between coal burning and ETS exposure.

Our outcome and exposure measures were determined by questionnaire alone, which is one of the major limitations of the study. Nonetheless, large epidemiological studies of respiratory health often rely on reports on recent symptom history because self-reported measures are cost efficient, practical and their repeatability is good [42,43]. Generally, respiratory symptoms have been reported consistently across populations [43]. To improve the quality of our self-reported outcomes we included audiovisual presentation of wheezing symptoms [15]. Because the temporal relationship between outcome(s) and exposure(s) can be difficult to determine in cross-sectional studies we focused on respiratory symptoms in the past 12 months to minimize recall bias. We did not use parents as source of information on child's symptoms. Some studies suggest that Chinese parents may deny or underreport child's symptoms or illnesses [44,45]. In addition, parents living in non-urban areas around Wuhan have lower educational level than parents living in urban areas [16], and their literacy level may be lower than their children attending middle school. Therefore, adolescents' reports on their own symptoms and health status may be more accurate than their parents'. Because children were answering in school about exposures in their home, we were not able to acquire very detailed information on exposure characteristics. Given that questionnaires have limited ability to quantify exposures, the possibility of exposure misclassification cannot be excluded. However, serious differential misclassification either of the exposures or outcomes is unlikely because health hazards of indoor air pollutants were not widely known among Chinese school children at the time when the survey was conducted [46].

Although urban air pollution has long been a major environmental concern in China, we do not believe that outdoor air pollution alone could explain the observed associations. Exposures to indoor air pollutants are likely to dominate the total exposure burden [47], especially among children, who spend much of their time inside the home [8]. In Chinese homes with coal stoves and smokers, not only levels of particulate matter, but also levels of many other air pollutants, including concentrations of SO_2_, often exceed the levels outdoors [2,3]. In Wuhan, where coal stoves are not usually vented via flue, concentrations of respirable particulate matter (291 μg/m^3^) and SO_2 _(173 μg/m^3^) can reach high levels indoors [2]. Concentrations of these pollutants have been found to be lower in ambient air. For example, a study investigating long-term air pollution in Wuhan estimated that the annual means for PM_2.5_, PM_10_, and SO_2 _in urban areas were 73 μg/m^3^, 129 μg/m^3^, and 73 μg/m^3^, respectively [48]. Because indoor air quality is influenced by infiltration of outdoor air, we cannot fully exclude possible confounding effects of ambient air pollution [1]. However, the effects of living area, as measured by school districts, were taken into account in our models, providing some control for differing air pollution levels in the study area.

The major strength of this study is that the public school system ensured a large and representative sample of rural, suburban, and urban populations in the Wuhan area. Our study is one of the few studies that have examined effects of major indoor pollutants in relation to children's respiratory health in mainland China [16,23-25].

## Conclusions

Coal burning and living with smokers contributed to persistent respiratory symptoms in this cohort of Chinese adolescents. Adverse effects of tobacco smoke in the home were seen despite the very low prevalence of maternal smoking. Even if exposure to residential coal burning declines in response to economic changes in China, the increasing prevalence in smoking augur an increase in children's exposure to environmental tobacco smoke. Because many men initiate smoking during adulthood, and the rate of quitting and desire to quit smoking are low [49], future prospects for children's health are worrisome. The rise in cigarette smoking in China is a growing public health concern, not only in the adult population but because its effects on children. Although rates of childhood asthma have remained low in China, common indoor air pollutants, coal and tobacco smoke, impair children's respiratory health.

## List of abbreviations

CI = confidence interval

CO = carbon monoxide

ETS = environmental tobacco smoke

OR = odds ratio

PM_10 _= particulate matter with an aerodynamic diameter less or equal to 10 μm

PM_2.5 _= particulate matter with an aerodynamic diameter less or equal to 2.5 μm

SO_2 _= sulfur dioxide

## Competing interests

The authors declare that they have no competing interests.

## Authors' contributions

Contributors: PMS analyzed the data and wrote the manuscript with input from all investigators. YL, JX and CL are key investigators for the data collection. ELA assisted with data collection, and GK assisted with data analysis. CAJ was involved in design of the study. SJL is the principal investigator and guarantor of the manuscript.
